# Icariside II in Neurological Disorders: Mechanistic Insights Into Alzheimer's Disease, Cerebral Ischemia and Parkinson's Disease

**DOI:** 10.1002/jbt.71093

**Published:** 2026-08-25

**Authors:** Ashutosh Solanki, Khushi Sharma, Jagrati Jain, Niraj Kumar Singh, Praveen T. Krishnamurthy

**Affiliations:** ^1^ Department of Pharmacology, College of Pharmacy JSS University Noida Uttar Pradesh India; ^2^ Anand College of Pharmacy, SGI Agra Uttar Pradesh India; ^3^ Division of Pharmacology, Institute of Pharmaceutical Research GLA University Mathura Uttar Pradesh India

**Keywords:** Alzheimer's disease, apoptosis, icariside II, neuroinflammation, neurological disorders, oxidative stress, Parkinson's disease

## Abstract

Icariside II (ICS II), a PDE5 inhibitor, is a flavonoid glycoside and primary metabolite of *icariin*, derived from the herb *Herba epimedii*, and exhibits promising neuroprotective potential in various pre‐clinical studies. The current review provides evidence that ICS II shows neuroprotective potential against various neurological disorders, mainly including Alzheimer's disease, Parkinson's disease and Cerebral ischemia through modulating neuroinflammation, oxidative stress, neural apoptosis, neurogenesis, mitochondrial and cognitive dysfunction. It regulates multiple signalling pathways including PI3K/Akt, Keap1/Nrf2, TLR4/MyD88/NF‐κB, cGMP/PKG/CREB, TGFB1/Smad, Wnt/β‐catenin signaling and BDNF/TrkB/CREB. The pre‐clinical evidence suggests that ICS II attenuates oxidative stress through increasing antioxidant enzymes SOD, GSH, catalase, and HO‐1, with decreasing MDA and lipid peroxidation. Neuroinflammation is suppressed by inhibition of pro‐inflammatory cytokines and down‐regulation of IL‐1β, IL‐6, TNF‐α, COX‐2, iNOS levels and up‐regulation of tight‐junction proteins like occludin, claudin‐5, ZO‐1. Apoptosis is regulated via altering PARP, Bcl‐2, Bax/Bcl‐2 ratio and reducing caspase‐3 activation. Mitochondrial dysfunction is ameliorated by restoring Complex I activity and increasing mitofusin‐1/2 expression and reducing mitochondrial fission factors. Collectively, these molecular mechanisms show promise for ICS II as a potential candidate in different in vivo and in vitro studies of neurological disorders.

## Graphical abstract

The graphical abstract represents the underlying neuroprotective potential of ICS II against Alzheimer's disease, Cerebral ischemia and Parkinson disease. ICS II downregulates ROS production, inflammation, mitochondrial dysfunction and apoptosis along with regulating their key signalling pathways.

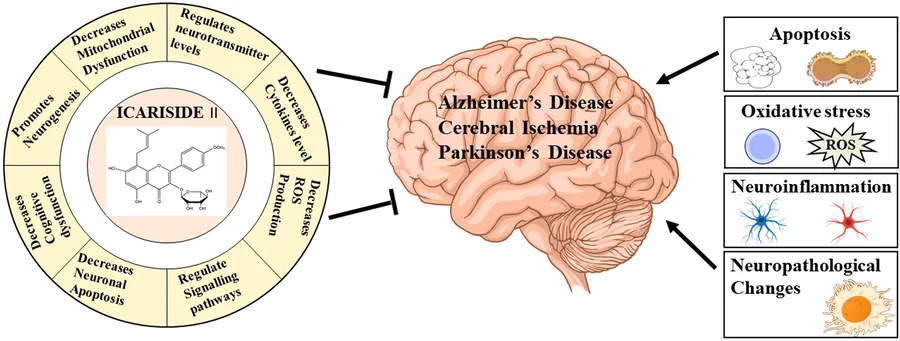

**Abbreviations**
AChE: Acetylcholinesterase
ADAM10: Disintegrin and metalloproteinase domain‐containing protein 10
AD: Alzheimer's Disease
Akt: Protein kinase B
APP: Amyloid precursor protein
Aβ: Amyloid‐beta
BACE: Beta‐site APP‐cleaving enzyme
Bax: Bcl‐2‐associated X protein
BBB: Blood‐brain barrier
Bcl‐2: B‐cell lymphoma 2
BDNF: Brain‐derived neurotrophic factor
CA1: Cornu Ammonis‐1
cAMP: Cyclic adenosine monophosphate
COX‐2: Cyclooxygenase‐2
CREB: cAMP response element binding protein
CTGF: Connective Tissue Growth Factor
DA: Dopamine
DAT: Dopamine Transporter
eIF2α: Inhibition of eukaryotic initiation factor α phosphorylation
ERK1/2: Extracellular signal‐regulated protein kinases
FOXO 3a: Transcription factor forkhead box O3 protein
GAP: Growth associated protein
GFAP: Glial fibrillary acidic protein
GPX4: Glutathione peroxidase‐ 4
GSH: Reduced Glutathione
GSK3β: Glycogen Synthase Kinase 3
hAMSCs: Human amniotic mesenchymal stem cells
HDAC‐2: Histone Deacetylase 2
HO‐1: Heme oxygenase‐1
HPA: Hypothalamic‐Pituitary‐Adrenal axis
IBA‐1: Ionized calcium‐binding adaptor molecule 1
IBO: Ibotenic acid
ICS II: Icariside II
IDE: Insulin‐degrading enzyme
IL‐1β: Interleukin‐1 beta
iNOS: Inducible nitric oxide synthase
IRS: Insulin receptor substrate
Keap 1: Kelch‐like ECH‐associated protein 1
lba1: Ionized calcium binding adapter molecule 1
LDH: Lactate dehydrogenase
LPS: Lipopolysaccharide
MAPK: Mitogen‐activated protein kinase
MAP‐2: Microtubule‐Associated Protein 2
MCAO: Middle Cerebral Artery Occlusion
MDA: Malondialdehyde
Mfn: Mitofusin
MMP: Matrix metalloproteinases
MPA: Mycophenolic acid
MPP: 1‐Methyl‐4‐phenylpyridinium
mRNA: Messenger Ribonucleic Acid
MyD88: Myeloid differentiation primary response protein 8
NEP: Neprilysin
NFκB RE: NF‐κB‐responsive elements
NF‐κB: Nuclear Factor Kappa‐light‐chain‐enhancer of Activated B Cells
NQO‐1: NAD(P)H:quinone oxidoreductase 1
Nrf2: Nuclear factor erythroid 2‐related factor 2
NSE: Neuron‐Specific Enolase
OGD/R: Oxygen glucose deprivation and reperfusion
OXPHOS: Oxidative Phosphorylation
PARP: Poly (ADP‐ribose) Polymerase
PC12: Pheochromocytoma cell line 12
PDE: Phosphodiesterase
PDE5: Phosphodiestrase type 5
PERK: PKR endoplasmic reticulum regulating kinase
PI3K: Phosphoinositide 3‐Kinase
PKG: Protein kinase G
PPAR: Peroxisome proliferator‐activated receptor
ROS: Reactive Oxygen Species
SAH: Subarachnoid hemorrhage
SIRT3: Sirtuin 3
Smad: Suppressor of Mothers against Decapentaplegic
SOD: Super oxide dimutase
STZ: Streptozotocin
TGF β1: Transforming growth factor Beta 1
TH: Tyrosine Hydroxylase
TIMP1: Metalloproteinase inhibitor 1
TLR4: Toll‐Like receptor 4
TNF‐α: Tumor necrosis factor alpha
TRAF6: Tumor Necrosis Factor Receptor Associated Factor 6
TrkB: Tyrosine protein kinase B
TUNEL: Terminal deoxynucleotidyl transferase dUTP nick‐end labeling assay
V1AR: Vasopressin V1a Receptor

## 1 Introduction

Progressive ageing of the global population and growing incidence of neurological disorders have emerged as major challenges to healthcare systems worldwide [1]. The Global Burden of Disease study in 2021 revealed that over 3 billion people in the world are suffering from different neurological disorders covering 1 in every 3 people affected which makes up to 43% of the world's population. This makes it the leading global disease burden ahead of cardiovascular disease [2]. Limited data on the prevalence, incidence, and severity of illnesses associated with neurological disorders are known in India. Globally, the causes have been limited to a small number of regional investigations [3]. Recent epidemiological studies show that the prevalence of these neurological disorders will increase substantially over the coming decades, affecting a larger proportion of the global population by 2030 [4]. The lack of effective curative therapies for neurological disorders remains a major challenge in modern medicine, which is partially driven by limited understanding of the underlying pathophysiology. The existing pharmacological therapies primarily provide only symptomatic relief rather than a complete cure, and are frequently associated with mostly of them are associated with adverse effects [5]. Despite their therapeutic benefits, currently available pharmacotherapeutic approaches are associated with several adverse effects which complicate treatment and the quality of life of patients. In recent years, researchers have focused on finding alternative approaches with safer and better therapeutic profiles. Plant‐derived bioactive compounds have gained considerable attention among researchers because of their broad spectrum of pharmacological activities, multitarget mechanisms of action, and favorable safety profiles, making them promising candidates for the management of neurological disorders [6, 7].

Icariside II (ICS II), a PDE5 inhibitor, is derived from an herb known as *Herba epimedii* (Yinyanghuo), family Berberidaceae, which has been widely traditionally utilised for generations for cardiovascular complications, osteoporosis and improving sexual function [8, 9]. The most prevalent and previously researched ingredient in *Herba epimedii* is icariin, which is converted into different metabolites, including Icariside‐I, Icariside‐II, Icaritin and desmethylicaritin [10, 11]. Research progress on ICS II has shown that ICS II exhibits pharmacotherapeutic effects, such as anti‐inflammatory, antioxidant, anti‐apoptotic, anti‐diabetic, anti‐cancer, and renal protective actions [12, 13]. It also shows a protective effect in rheumatic disorders and strengthens bones and muscles [14]. Recent research studies have identified ICS II as a potential therapeutic candidate in the treatment of neurological disorders [15]. The current review comprehensively summarizes the neuroprotective potential and underlying molecular mechanisms of ICS II across various in vitro and in vivo models of neurological disorders Table 1.

**Table 1:** Representative of doses of Icariside II, animal and cell culture used, screening model and proposed mechanism against neurological disorders.

S. No.	Neurological Disorder	Name of the compound/Formulation, Duration of treatment	Study design/Screening Model	Site of Administration With the Agent used for disease induction	Animals Used/cell culture/Patient	Molecular Mechanism	Reference
1	Alzheimer's Disease	In vivo: Icariside II (3, 10 mg/kg), 15 days In vitro: Icariside II (0.01%), dissolved in DMSO at 10 nM as a stock solution.	In‐vivo‐Aβ25‐35‐induced cognitive Dysfunction. In vitro neuronal‐like PC12 cells injury model	In vivo‐ Aβ_25‐35_, ICV In‐vitro‐PC12 cells treated with Aβ_25–35_, 20 μM	Sprague‐Dawley rats In‐vitro‐PC12 cells	Increases the expression of BDNF and TrkB and activates CREB phosphorylation Decreases the levels of PDE5 and cGMP in hippocampus. Attenuates Aβ_25–35‐_induced apoptosis through regulating BDNF/TrkB/CREB signal pathway Decreases ROS generation and mitochondrial dysfunction	[16]
2	Cognitive Dysfunction	Icariside II (4, 6, and 16 mg/kg), 20 days	In‐vivo‐ Ibotenic acid‐induced cognitive impairment	Ibotenic acid (2 μg/μL), Hippocampal injection,	Sprague‐Dawley rats	Suppress Bax/Bcl‐2 levels and caspase‐3 levels Downregulates calbindin protein and MAPK (p38, ERK1/2 and JNK) pathway	[9]
3	Alzheimer's disease	Icariside II (20 mg/kg), 15 Days	In‐vivo‐ Aβ25‐35‐induced cognitive Dysfunction.	Bilateral hippocampal injection of 5 μL Aβ_25–35_ (2 μg/μL)	Adult male Sprague‐Dawley rats	Decreases the expression levels of IL‐1β, TNF‐α, COX‐2, iNOS mRNA Decreases the ratio of apoptotic markers Bax/Bcl‐2 and caspase‐3. Suppresses microglial and astrocytic activation	[17]
4	Alzheimer's disease	ICS II (5, 10, and 20 μM), 1 h	In vitro lipopolysaccharide (LPS)‐induced neuroinflammation.	Cells were treated with LPS (1 mg/mL)	Primary astrocytes extracted from neonatal brains of Sprague‐Dawley rats	Mitigates the level of TNF‐α, IL‐1β, iNOS, COX‐2 Inhibits IκB‐α degradation and NF‐κB activation Decreases the levels of β‐APP and BACE1	[18]
5	Alzheimer's disease	Icariside II (3 or 10 mg/kg), 21 days	In‐vivo‐ Streptozotocin induced cognitive dysfunction	STZ (1.5 mg/kg), bilaterally into ventricle	Male Sprague‐Dawley	Enhances the expressions of IL‐1β, TNF‐α, COX‐2 and TGF‐ β1 levels Downregulates IkB‐α degradation and NF‐ κB activation Decreases β‐APP, BACE1 levels and promotes Aβ degradation	[19]
6	Cognitive Dysfunction	Icariside II (4, 8, and 16 mg/kg), 28 days	In‐vivo‐ CCH‐induced cognitive dysfunction	N/A	Sprague‐Dawley rats	Mitigates the expression of β‐APP, BACE1 Upregulates the expression of disintegrin, ADAM10 and IDE Enhances the levels of BDNF, TrkB, Akt and CREB phosphorylation Upregulates the expression of PPARα and PPARγ	[20]
7	Alzheimer's disease	Icariside II (10 and 30 mg/kg), 3 months	In vivo Screening model‐ N/A	N/A	APP/PS1 transgenic mice and their littermates, WT mice	Upregulates disintegrin, ADAM10, downregulates APP and BACE1 and PPARγ degradation Inhibits p‐PERK/p‐eIF2α phosphorylation	[21]
8	Chronic Hydrocephalus and Cognitive Decline	Icariside II (1,5,10 mg/kg), 21 days	In vivo SAH Model	N/A	Sprague‐Dawley rats	Improves long‐term cognitive function and inhibits the TGF‐β1/Smad/CTGF signalling pathway	[22]
9	Cognitive Dysfunction	Icariside‐II (2.5, 5, 10 mg/kg), 28 days	In vivo study Screening model‐ N/A	N/A	APP/PS1 mice	Increases the expression of Wnt/β‐catenin. Downregulates mitochondrial fission by upregulating Mfn 1, Mfn 2 while downregulating Fis 1, Mff, p‐Drp 1	[23]
10	Alzheimer's Disease	Icariside II (0.25, 0.5, 1 μM), 4 h	In‐vitro study Screening model‐N/A	NSCs were infected with APP‐GFP lentiviral vector or GFP lentiviral vector	Hippocampal NSCs obtained from C57BL/6 mice	Upregulates the expression of Wnt/β‐catenin pathway.	[24]
11	Cerebral ischemia/reperfusion Injury	Icariside II (16 mg/kg), 3 Days	In‐vivo‐ Middle Cerebral Artery Occlusion Model	Carotid artery occlusion	Male Sprague–Dawley rats	Increases the expression of TIMP1 and tight junction proteins‐ occludin, claudin 5, and ZO‐1 Decreases the expression of MMP2/9 and apoptotic markers like‐Bax/Bcl‐2 ratio and caspase 3 level	[25]
12	Cerebral ischemia/reperfusion Injury	Icariside II (20 mg/kg), 7 Days	In‐vivo‐ Middle Cerebral Artery Occlusion Model	N/A	Sprague‐Dawley rats	Activates OXPHOS/NF‐κB/ferroptosis axis Upregulates the expression of Nrf2 signaling pathway	[26]
13	Cerebral ischemia/reperfusion Injury	Icariside II (12.5, 25, 50 μM), 24 h	In‐vivo‐ OGD/R‐induced primary hippocampal neuron injury.	Primary hippocampal neurons were extracted from newly born rats and cultured in DME/F‐12 medium	Sprague‐Dawley rats	Decreases the expression of apoptotic markers like‐Bax/Bcl‐2 ratio and caspase 3 level Upregulates cGMP levels and PKG/CREB/BDNF/TrkB signaling pathway Increased the phosphorylation of CREB, BDNF, and TrkB	[27]
14	Cerebral ischemia/reperfusion Injury	Icariside II (12.5, 25, and 50 μM), 24 h	In‐vitro‐ OGD/R‐induced PC12 cell oxidative injury	N/A	PC 12 cells	Increases Bcl‐2 while decreasing Bax and caspase‐3 expressions Upregulates the expression of Nrf2/SIRT3 signalling pathway	[28]
15	Cerebral ischemia/reperfusion Injury	Icariside II (20 mg/kg)	In‐vivo‐ Bilateral Common Carotid Arteries Occlusion Model	N/A	Mongolian gerbils	Inhibits leukocyte adhesion and albumin leakage Down‐regulates the expression of Bax and caspase‐3 while up‐regulates Bcl‐2 expression. Decreases MDA content in hippocampus	[29]
16	Cerebral ischemia/reperfusion Injury	Icariside II (4, 8, and 16 mg/kg^−1^), 3 Days	In vivo Middle Cerebral Artery Occlusion Model	N/A	Sprague‐Dawley rats	Increases cGMP level, PKG activity and inhibits neuronal autophagy alters PKG/GSK‐3β/autophagy axis	[30]
17	Cerebral ischemia‐reperfusion injury	Icariside II (10 or 30 mg kg^−1^), 3 days	In vivo Middle Cerebral Artery Occlusion Model	N/A	Sprague‐Dawley rats	Decreases IL‐1 β, TGF‐β1, IkB‐ α, NF‐kB. Increases the expression of PPAR α and PPAR β	[31]
18	Cerebral ischemia‐reperfusion injury	Icariside II (5, 10 and 20 mg/kg), 7 days	In‐vivo‐ Middle cerebral artery occlusion model	N/A	Sprague‐Dawley rats	Decreases ROS content, further increasing SOD, GSH‐Px, catalase levels and decrease in MDA levels Enhances the expression of Nrf2/HO‐1 signaling pathway	[32]
19	Neuropathic Pain	Icariside II (5, 10 μM), 7 days	In silico study Screening model‐N/A	N/A	Docking‐based study	Alleviates inflammation and neuropathic pain through inhibiting the biochemical interactions between USP5 and Cav3.2. Additionally, blocking Cav3.2 channels	[33]
20	Neuropathy	Icariside II (5 mg/kg), 12 weeks	In vivo‐STZ‐induced diabetic model	Streptozotocin (60 mg/kg), i.p.	Sprague‐Dawley rats	Attenuates the diabetes‐related impairment and major pelvic ganglion neuropathy in rats Increases the expression of NGF and nNOS	[34]
21	Neuro inflammation	Icariside‐II (3, 10 mg/kg), 7 Days	In vivo lipopolysaccharide‐induced neuroinflammation	Lipopolysaccharide (10 μg/μL), ICV injection	Male Sprague‐Dawley rats	Inhibits IBA‐1 of microglia and GFAP Downregulates the expression of IL‐1β, TNF‐α, COX‐2. Decreases the levels of TLR4. MyD88 and TRAF6	[10]
22	Parkinson's Disease	Icariside II (1, 3, 10 μmol/L), 21 days	In vitro study Screening model‐ N/A	N/A	Human amniotic mesenchymal stem cells were cultured in DMEM/F12 medium	Increases the protein expression levels of NSE, MAP‐2, and TH and Triggers hAMSCs to differentiate into dopaminergic neuron‐like cells through involvement of PI3K signaling pathway.	[35]
23	Neuron‐ like cell proliferation	Icariside II (25, 50, and 100 μM), 72 h	In vitro study Screening model‐ N/A	N/A	PC12 cells were cultured in DMEM medium	ICS II promotes proliferation, Increases NO level via activating AKT and suppressing NO degradation via inhibiting PDE5. Upregulates the expression of cGMP/PKG pathway	[36]
24	Neurotoxicity	Icariside II (10, 30 mg/kg), 4 weeks	In‐vivo‐ METH‐abuse mice model	Methamphetamine (1‐5 mg/kg), i.p.	C57BL/6 J mice	Increases TH, DAT and DA levels and decreases GFAP, Iba1, and α‐Syn levels Upregulates the expression of the Keap1‐Nrf2 signaling pathway	[37]
25	Cerebral hypoperfusion	Icariside II (4, 8 mg/kg), 12 weeks	In vivo CCH Model	N/A	Sprague‐Dawley rats	Upregulates the expression of proteins, GAP‐43 and MPA‐2 Reduce Noga in the CA1 area of hippocampus. Additionally, promoting learning and memory ability	[38]
26	Parkinson's Disease	Icariside II (12.5, 25 and 50 μM), 24 h	In vitro MPP + ‐Induced Parkinson's Disease	Cells were treated with 2 mM of MPP^+^	Human neuroblastoma SK‐N‐SH cell line	Decreases apoptosis, oxidative stress, and ferroptosis in MPP^+^‐treated cells. Activates Keap1/Nrf2/GPX4 signaling pathway	[39]
27	Parkinson's Disease	Icariside II (0, 12.5, 25 and 50 µM), 24 h	In vitro MPP + ‐induced Parkinson's Disease	Cells were treated with 2 mM of MPP^+^	Human neuroblastoma SK‐N‐SH cells	Alleviates DNA damage and mitochondrial dysfunction in MPP + ‐induced SK‐N‐SH cells Decreases the expression of HDAC2 protein	[40]
28	Schizophrenia	Icariside II (1, 3, and 10 mg/kg), 1 h	In‐vivo‐MK‐801‐induced ‐like symptoms	MK‐801 (0.2 mg/kg), i.p.	ICR male mice	Inhibits cAMP production and upregulates Akt/GSK‐3β signaling pathway Regulates neuronal GPCRs through D3R and V1AR action	[41]

## 2 Data Sources and Search Strategy

Relevant research studies were collected through an electronic search on Google Scholar, PubMed and ScienceDirect databases. The keywords used for search was “Icariside II” paired with “Neurodegenerative disorders”, “Alzheimer's disease”, “Parkinson's disease”, “Cerebral ischemia”, “Neuropathic pain”, “Neuroinflammation”, “Neurotoxicity”, “Cognitive dysfunction”, “PI3K/Akt”, “Keap1/Nrf2”, “TGFB1/Smad”, “Wnt/β‐catenin” and “TrkB/CREB”. A comprehensive literature search was conducted using these terms to collect relevant research and review studies for the present study. Through this search strategy, we identified 198 research articles, and among them, 28 research studies published between 2015 and 2023 were selected to be included in the study. We applied the inclusion criteria, such as pre‐clinical experimental studies, in vivo, in vitro and in silico, English language articles. The selected articles were analyzed thoroughly to extract scientific evidence, and the articles that were outside the scope of this study were excluded from the present study.

## 3 Sources and Chemistry of Icariside II

**Figure 1:**
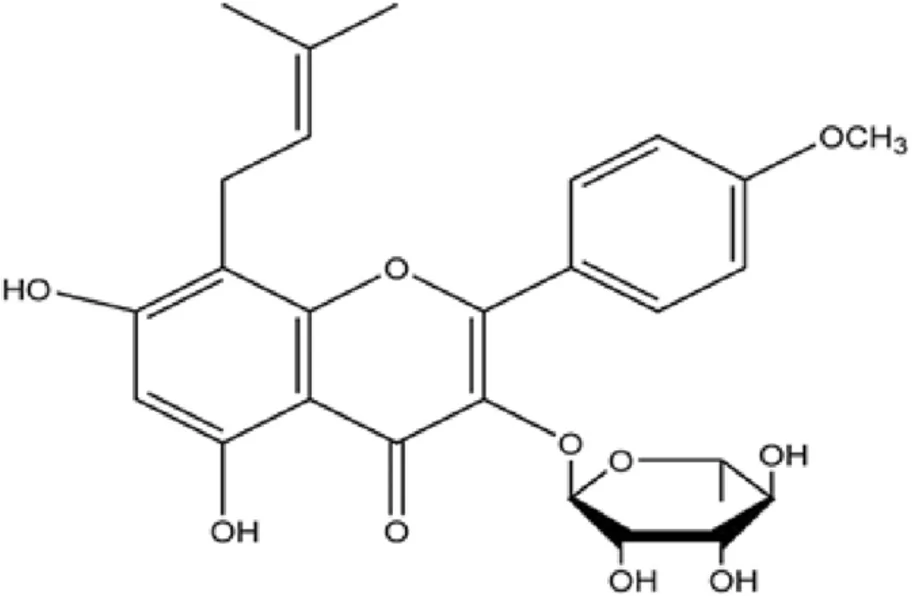
Chemical structure of Icariside II.

ICS II is an active flavonoid found in the medicinal herb *Herba epimedii* (commonly known as *Yin‐yang‐huo* in China, Horny goat weed in English) and *Cortex periplocae*, commonly known as *Xiang Jia Pi* in China, is made up of dry roots of the herb *Periploca sepium Bunge* [42]. According to the Chinese pharmacopoeia, *Herba Epimedii* mainly consists of the dried aerial portion of *E. pubescens Maxim, E*. *koreanum Nakai, E*. *sagittatum Maxim*, and *E. devidii* [43]. ICS II is a flavonol glycoside that is *3,5,7‐trihydroxy‐4’‐methoxy‐8‐prenylflavone*. In the structure of ICS II (Shown in Figure 1), one glucose moiety is removed from icariin's C7 position. The chemical formula (IUPAC) of ICS II is *(5,7‐dihydroxy‐2‐(4‐methoxyphenyl)−8‐(3‐Methylbut‐2‐enyl)−3‐[(2S,3 R,4 R,5 R,6S)−3,4,5‐Trihydroxy‐6‐methyloxan‐2‐yloxychromen‐4‐One)*. It has 514.52 g/mol molecular weight and the chemical formula is C_27_H_30_O_10_. It shows poor water solubility in both water and organic solvents [44].

## 4 Pharmacokinetic Profile of Icariside II

**Figure 2:**
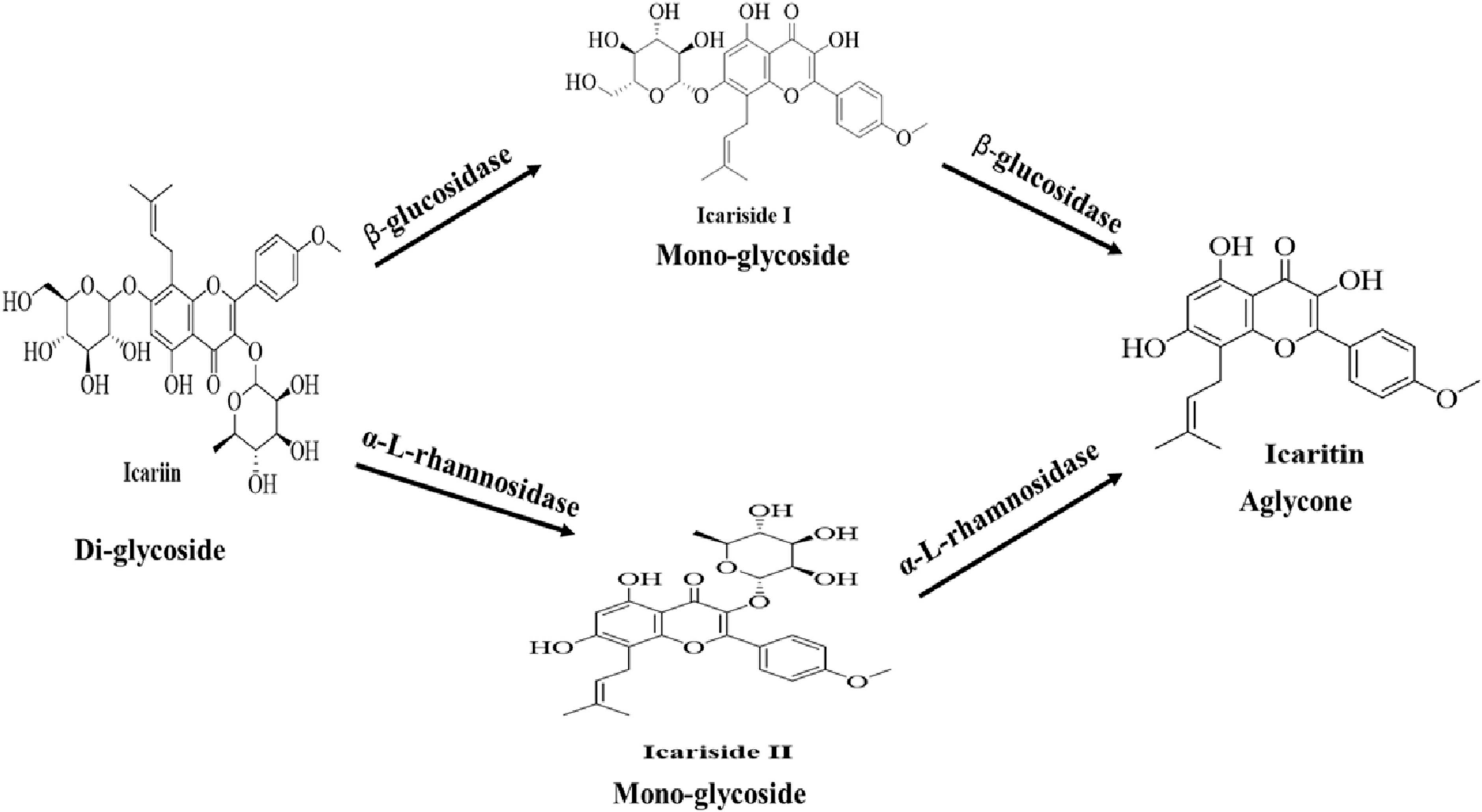
Schematic representation of the intestinal metabolism of icariin after oral administration. Icariin (di‐glycoside) is consecutively hydrolyzed by intestinal β‐glucosidase and α‐L‐rhamnosidase to yield the mono‐glycosides, icariside I and icariside II, followed by further de‐glycosylation to form the aglycone metabolite icaritin.

To address the pharmacokinetic properties, the major limitation is that research studies focusing on the pharmacokinetics of ICS II are limited; studies often investigate its relationship with icariin, a precursor compound [45]. Research studies suggest that after oral administration of icariin to rats, a significant portion (91.2%) is metabolized into ICS II by intestinal flora, resulting in higher plasma concentrations of ICS II compared to icariin [46]. This suggests that oral administration may be a more effective route for delivering ICS II. However, intravenous administration shows a different pattern, with lower ICS II concentrations compared to icariin. This difference highlights the importance of first‐pass metabolism and intestinal conversion in the pharmacokinetics of ICS II (Shown in Figure 2) [47]. Rapid tissue distribution occurs in rats, with the kidney, lung, and liver exhibiting the highest first concentrations, followed by muscle, heart, bone, spleen, and plasma exhibiting moderate distributions. Small amounts were detected in the testes [48]. ICS II is primarily produced through the biotransformation of icariin by intestinal flora [49]. This metabolic pathway involves the enzymatic hydrolysis of the glucose moiety from icariin, a diglycoside, by α‐L‐rhamnosidase, yielding ICS II. The hydrolysis catalyzed by β‐glucosidase produces Icariside I from Icariin. Notably, the metabolic process of ICS II progresses and results in additional biochemical transformations that yield icaritin as a by‐product [50, 51]. The preclinical studies included in this review consistently demonstrate the neuroprotective role of ICS‐II across various experimental models of neurological disorders, but none of these studies have evaluated its Blood‐Brain Barrier (BBB) penetration, brain distribution kinetics, and bioavailability in the CNS. Except [28], which reported that ICS II treatment reduces BBB disruption following cerebral ischemia/reperfusion (I/R) injury. Therefore, further pharmacokinetic and biodistribution investigations are required to quantitatively establish its BBB permeability, brain distribution and therapeutic exposure within the CNS.

## 5 Overview of the Therapeutic Potential of Icariside II Against Neurological Disorders

### 5.1 Role of Icariside II on Alzheimer's Disease

Alzheimer's disease (AD) is the progressive neurodegenerative disorder characterised by extracellular accumulation of a specific protein, amyloid‐beta (Aβ) peptide fibrils, causing neuro‐inflammation, cognitive dysfunction and loss of memory [52, 53]. Further, it leads to increased production of pro‐inflammatory cytokines and additional dysfunction of the hypothalamic‐pituitary‐adrenal (HPA) axis as well as the glutaminergic and cholinergic systems [54]. According to reports, 46.8 million individuals worldwide suffered from dementia in 2015, and the number of AD patients is increasing by more than 11 million/year. This puts a significant strain on society [55]. The characteristic clinical traits of AD lead to a progressive malfunction in the brain, which affects memory and spatial learning [56]. In AD, inflammatory mediators elevate beta‐secretase activity and promote the production of Aβ and the expression of amyloid precursor protein (APP). The progression of AD disrupts neurotransmitter and neurohormonal homeostasis, through elevated levels of ROS, AChE and cortisol [57, 58].

Various research studies revealed that ICS II possesses a therapeutic role in the management of AD. In an in vivo and in vitro study, it was found that ICS‐II attenuates cognitive dysfunction and neuronal cell injury induced by Aβ_25–35_ in rats. It decreases PDE5 and cGMP levels. Additionally, ICS II treatment reduces both intracellular and mitochondrial ROS production and restores mitochondrial dysfunction in PC12 cells. In vivo and in vitro, both results suggest that it also inhibits neuronal apoptosis by upregulating the BDNF/TrKB/CREB signalling pathway in the hippocampus [16]. In another study, ICS II attenuates Aβ‐induced neuronal damage and apoptosis in the hippocampal region. It possesses the ability to block the initiation of microglia and astrocytes, suppresses the increase of the Bax/Bcl‐2 ratio and caspase‐3 activation caused by Aβ, and downregulates the expression of IL‐1β, TNF‐α, COX‐2, and iNOS mRNA [17]. In another study, it was revealed that Pre‐treatment with ICS II reduced the levels of astrocyte‐derived IL‐1β, TNF‐α, COX‐2 and iNOS in a dose‐dependent manner. It also inhibited the NF‐κB activation and IκB‐α degradation and reduced the levels of BACE1, APP, and Aβ1‐40 in the astrocytes [18]. It has been stated that ICS II attenuates cognitive dysfunction, neuronal death and decreases the levels of Aβ1‐42 and PDE5 in the hippocampus, and it downregulates the expression of APP, BACE1, TNF‐ α, COX‐2, TGF‐β1, NFκB and upregulates NEP [19]. Furthermore, it was documented that ICS II administration improves learning and memory function, prevents neuronal degeneration and plaque formation. It promotes the non‐amyloidogenic APP cleavage process by elevating the expression of a disintegrin and ADAM10. It also inhibits the amyloidogenic APP processing pathway by suppressing the expression of APP and BACE1. ICS II treatment reduces the degradation of PPARγ, phosphorylation of PKR, endoplasmic reticulum regulating kinase and eukaryotic initiation factor‐α [21]. In an in vitro study, researchers found that ICS II treatment decreases apoptosis and promotes proliferation and neuronal differentiation of APP overexpressing neural stem cells. It causes the activation of the Wnt/β‐catenin signalling pathway along with inhibition of GSK‐3β [24]. In a study, it was shown that ICS‐II attenuates IBO‐induced cognitive dysfunction and neuronal death through increasing the expression of calbindin and downregulating the expression of the MAPK Signalling pathway. ICS‐II treatment decreases the levels of the Bax/Bcl‐2 ratio and caspase‐3 [9]. Furthermore, it was found that ICS II improves long‐term cognitive dysfunction and decreases subarachnoid fibrosis by downregulating the expression of the TGF‐β1/Smad/CTGF signalling pathway [22]. Furthermore, it was also discovered that ICS II decreases cognitive dysfunction and neuronal injury in the hippocampus. It down‐regulates the expression of APP and BACE1, while upregulates the expression of Disintegrin, ADAM 10 and IDE. Additionally, ICS II treatment causes activation of the BDNF/TrKB/CREB signalling pathway and increases the levels of PPAR‐α and PPAR‐ γ [20]. Another study found that ICS II attenuates cognitive dysfunction and enhances neurogenesis in the hippocampus. ICS II treatment decreases Mitochondrial fission by upregulating the levels of Mfn 1 and Mfn 2, and downregulating the levels of mitochondrial fission factor, mitochondrial fission 1 protein and phosphorylated dynamin‐related protein 1. Further, it upregulates the expression of the Wnt/β‐catenin signalling pathway [23]. In summary, the findings suggest that ICS II improves the symptoms in the management of AD.

**Figure 3:**
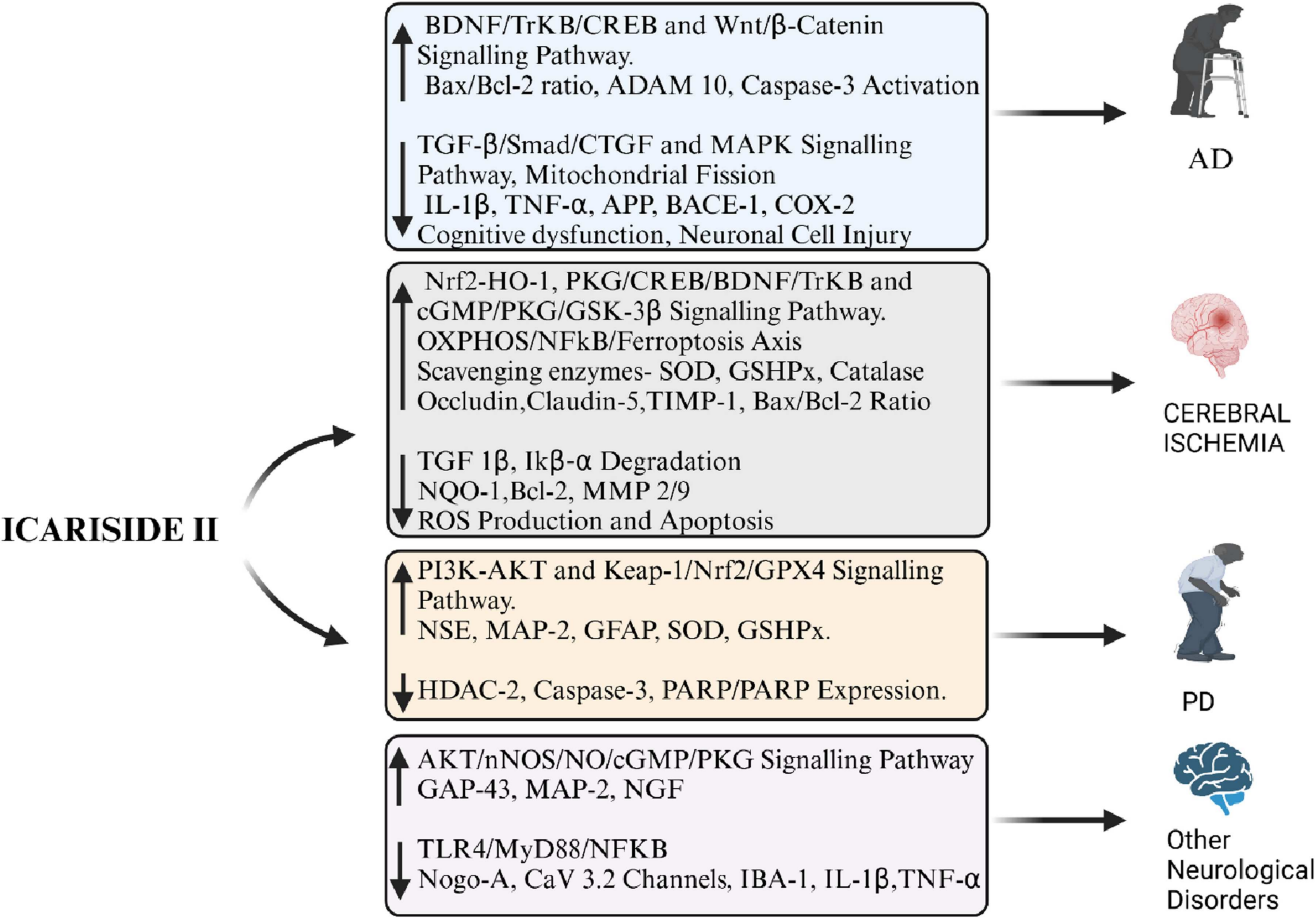
Schematic representation of different cellular and molecular mechanisms involved in the neuroprotective effects of Icariside II against Alzheimer's disease, Cerebral ischemia, and Parkinson's disease.

Overall, the available preclinical evidence consistently demonstrates that ICS II exerts neuroprotective effects against AD through modulating multiple mechanisms, including attenuation of amyloid pathology, suppression of neuroinflammation and oxidative stress, inhibition of neuronal apoptosis, enhancement of neurogenesis, and modulation of mitochondrial function, which is regulated by altering key signalling pathways, such as BDNF/TrkB/CREB, Wnt/β‐catenin, PPARγ, NF‐κB, GSK‐3β and TGF‐β/Smad/CTGF (Shown in Figure 3). These evidence supports that ICS II acts as a multi‐target therapeutic agent against AD. Despite these promising preclinical findings, several limitations should be considered when interpreting the available research evidence. Most studies have been conducted using rodent models or in vitro cell lines, particularly Aβ‐induced models, which primarily reproduce amyloid‐related pathology but does not cover the complex and multifactorial pathology of human AD, including tau pathology, chronic neurodegeneration, and age‐associated disease progression.

### 5.2 Role of Icariside II in Cerebral Ischemia

Cerebral Ischemia/reperfusion injury is a neurological condition, characterized by rupture or obstruction of cerebral arteries, resulting in interruption of cerebral blood supply [59, 60]. Reduced blood flow to the brain leads to a deficiency of oxygen supply and causes impairment in the oxygen‐deprived region of the brain [61]. Thrombosis is one of the underlying causes of ischemic stroke, as it interrupts cerebral blood flow. This interruption leads to brain tissue necrosis and neuronal injury, along with mitochondrial dysfunction, oxidative stress, cytokine production, inflammation, and subsequent tissue damage [62, 63]. Cerebral Ischemia/reperfusion injury is the second leading cause of death globally and has a high disability rate as well as a high socioeconomic cost to society and the affected individuals [64]. The only approved medication to treat ischemic stroke is recombinant tissue plasminogen activator, a thrombolytic drug with a limited therapeutic window and serious adverse effects [65, 66].

Various studies suggest that ICS II possesses a therapeutic role in the management of cerebral ischemia. In a study, it was found that ICS II treatment decreases ROS production and MDA levels. It increases the content of SOD, GSH‐Px and catalase. Additionally, it activates the Nrf2/HO‐1 Signalling Pathway [32]. Furthermore, it was discovered that ICS II possesses a neuroprotective effect in the MCAO rat model. The effect is achieved by PPAR‐mediated regulation of NF‐κB signalling, which results in decrease in the production of TGF‐1β and IL‐1β. Additionally, ICS II prevents IκB‐α degradation, thereby inhibiting NF‐κB activation [31]. Another study signified that ICS II increases tight junction proteins‐occludin, claudin 5, and ZO‐1 and TIMP1 expression, while decreasing the expression of MMP2/9. Molecular docking indicated that ICS II can bind directly to MMP2 and MMP9. Further, it decreases apoptosis via increasing the Bax/Bcl‐2 ratio and decreases BBB disruption, regulated by the Caspase‐3 dependent pathway [25]. Moreover, it was observed that ICS II treatment mitigates hippocampal neuronal dysfunction through enhancing neuronal viability and decreasing cellular LDH release. Additionally, it upregulates cGMP levels and PKG activity. ICS II activates the PKG/CREB/BDNF/TrkB signalling pathway and possesses neuroprotective effect against OGD/R‐induced primary hippocampal neuronal injury [27]. In another study, Researchers found that ICS II attenuates cerebral microcirculatory disturbance, neuronal loss and CA1 region injury in the hippocampus and reduces leukocyte adhesion, albumin leakage and increases the RBC velocity in cerebral venules. ICS II downregulates the expression of Bax and cleaved Caspase‐3 while upregulating Bcl‐2 expression. Additionally, it also increases Complex I activity and decreases MDA production [29]. In an in vitro study, it was found that ICS II treatment ameliorates OGD/R‐induced PC12 cell injury through increasing Cell viability and decreasing LDH leakage. It inhibits apoptosis and overproduction of mitochondrial ROS. The effect is mediated by downregulating the expression of HO‐1, NQO‐1, and cleaved caspase‐3 levels, Bcl‐2 protein and upregulating Bax, Keap1. Further, it activates the Nrf2/SIRT3 signalling pathway [28]. According to a study, it was found that ICS II reduces cerebral I/R injury by inhibition of PDE5 and excessive neuronal autophagy. The protective effect is mediated via the cGMP/PKG/GSK‐3β signalling pathway [30]. Furthermore, it was reported that ICS II protects against neurological impairments and improves long‐term recovery with BBB permeability following ischemic stroke. It inhibits astrocyte activation through the Nrf2‐regulated OXPHOS/NF‐κB/ferroptosis axis and produces cytoprotective effect on astrocyte cultures [26].

In summary, it can be concluded that ICS II shows neuroprotective potential against different preclinical models of cerebral ischemia/reperfusion injury through its ability to modulate oxidative stress, neuroinflammation, apoptosis, mitochondrial dysfunction, blood‐brain barrier (BBB) integrity, autophagy, and ferroptosis. The involvement of multiple signaling pathways, including Nrf2/HO‐1, Nrf2/SIRT3, cGMP/PKG/GSK‐3β, PKG/CREB/BDNF/TrkB, PPAR/NF‐κB, and OXPHOS/NF‐κB/ferroptosis, indicates that ICS II exerts neuroprotective effect against different preclinical models of Cerebral ischemia/reperfusion injury (Shown in Figure 3). Although the available evidence demonstrates the neuroprotective potential of ICS II, several limitations should be considered. Most research studies have been conducted on acute rodent ischemia models and cell culture preparations, which do not fully replicate the complexity of human ischemic stroke, particularly with ageing and coexisting conditions like hypertension and diabetes, and delayed therapeutic intervention. Considerable variability in experimental design, including differences in ischemia induction methods, ICS II dosage, treatment schedules, routes of administration, and outcome measures, also limits direct comparison across studies and prevents determination of an optimal therapeutic regimen.

### 5.3 Role of Icariside II in Parkinson's Disease

Parkinson's disease (PD) represents the second most prevalent neurodegenerative disease after AD [67]. It can be pathologically explained by loss of dopaminergic neurons in the substantia nigra pars compacta region and nondopaminergic neurons, such as noradrenergic, serotonergic, and cholinergic neurons [68]. Clinical motor symptoms of PD include tremor, bradykinesia, dysphagia, and postural instability. While some patients also experience non‐motor symptoms like hyposmia, depression, psychosis, hypotension and constipation. α‐synuclein aggregation, Neuroinflammation, oxidative stress, and free radicals are some key factors which plays crucial role in the pathogenesis of PD [69, 70]. Some studies have explored the therapeutic potential of ICS II in PD. In a study of PD, researchers revealed that ICS II treatment triggers the differentiation of hAMSCs into dopaminergic neuron‐like cells, which is mediated by activation of the PI3K Signalling pathway. Additionally, it promotes nerve‐related protein expression‐ MAP‐2, NSE, GFAP, and TH and increases the dopamine release in hAMSCs [35]. In another study, it was found that ICS II inhibit cell apoptosis through decreasing the levels of caspase‐3, proapoptotic Bax, cleaved PARP/PARP expression and increasing anti‐apoptotic Bcl‐2 expression. Additionally, it increases the level of SOD, GSH‐px, whereas decreases the level of pro‐oxidant MDA. It was found that ICS II regulates the above effect through activating the Keap1/Nrf2/GPX4 signalling pathway [39]. Furthermore, in another study, it was observed that ICS II attenuates DNA damage and mitochondrial dysfunction and protects dopaminergic neurons from MPP + ‐induced neurotoxicity. Additionally, it also downregulates the expression of HDAC2 [40]. The research findings suggest that Icariside II (ICS II) exerts promising neuroprotective effects in PD by targeting multiple pathological processes, including oxidative stress, mitochondrial dysfunction, apoptosis, and dopaminergic neuronal loss. The reported activation of PI3K and Keap1/Nrf2/GPX4 signaling pathways, together with the inhibition of HDAC2, indicates that ICS II exerts neuroprotective effects through the coordinated modulation of multiple interconnected molecular pathways implicated in PD (Shown in Figure 3). Moreover, the ability of ICS II to promote the differentiation of human amniotic mesenchymal stem cells into dopaminergic neuron‐like cells highlights its potential role in neuronal regeneration in addition to neuroprotection. Despite its promising therapeutic potential, evidence supporting the therapeutic role of ICS II in PD remains limited. Most studies have relied on preclinical models that only partially mimic human PD, while methodological variability and insufficient data on pharmacokinetics, blood–brain barrier permeability, safety, and long‐term efficacy continue to limit its translational potential. Therefore, future studies should employ standardized experimental designs, validate findings in chronic and transgenic animal models that better mimic human PD, and ultimately conduct well‐designed clinical trials to determine the therapeutic efficacy and safety of ICS II in patients with PD.

### 5.4 Role of Icariside II in Other Neurological Disorders

Numerous research studies have documented that ICS II possesses therapeutic role in different ND.

#### 5.4.1 Neuroinflammation

ICS II treatment suppresses microglia activation by decreasing IBA1 levels and astrocyte activation by GFAP levels. Additionally, ICS II decreases neuroinflammation by downregulating the expression of the TLR4/MyD88/NF‐κB pathway, which leads to decrease in inflammatory markers like IL‐1β, TNF‐α, and COX‐2 [10].

#### 5.4.2 Chronic Cerebral Hypoperfusion

In a study of Chronic cerebral hypoperfusion, ICS II treatment increases the expression of neuronal axon regeneration‐related proteins, GAP‐43, MAP‐2, while decreasing Nogo‐A levels in the CA1 region of the hippocampus [38].

#### 5.4.3 Diabetic Neuropathy

ICS II administration ameliorates diabetes associated dysfunction of corpus cavernosum and major pelvic ganglion neuropathy through increasing the expression of NGF and nNOS [34].

#### 5.4.4 Neuron‐Like Cell Proliferation

ICS II treatment promotes the proliferation of neuron‐like, highly differentiated rat PC12 cells by upregulating AKT, an upstream modulator of nNOS, leading to increased nitric oxide production. Additionally, it also upregulates cGMP content and PKG activity and collectively activates the AKT/nNOS/NO/cGMP/PKG signalling pathway [36].

#### 5.4.5 Neurotoxicity

ICS II attenuates METH‐induced neurotoxicity, decreases neuronal loss and improves PD‐like behavioural impairment. It increases the expression of TH, DAT, and DA, while it decreases GFAP, IBA1, and α‐synuclein in the region of the striatum. Additionally, it also activates the Keap1‐Nrf2 signaling pathway [37].

#### 5.4.6 Neuropathic Pain

ICS II efficiently blocked Cav3.2 channels while also reducing the biochemical connections between USP5 and Cav3.2 when ICS‐II binds to the Cav3.2 interaction area as well as the cUBP domain. It was expected that ICS‐II would interact with residues near the fenestrations of the Cav3.2 channel. Consequently, explaining the observed blocking action. ICS‐II exerts analgesic effects by acting on the Cav3.2 channel [33].

#### 5.4.7 Schizophrenia

ICS II potentiates dopaminergic function by acting as a selective D3R agonist; it inhibits cAMP formation and regulates the expression of the Akt/GSK‐3β signalling pathway. Further, it decreases anxiety and improves memory performance through the V1AR antagonist action [41].

### 5.5 Mechanistic Integration of ICS II‐Mediated Neuroprotection

**Figure 4:**
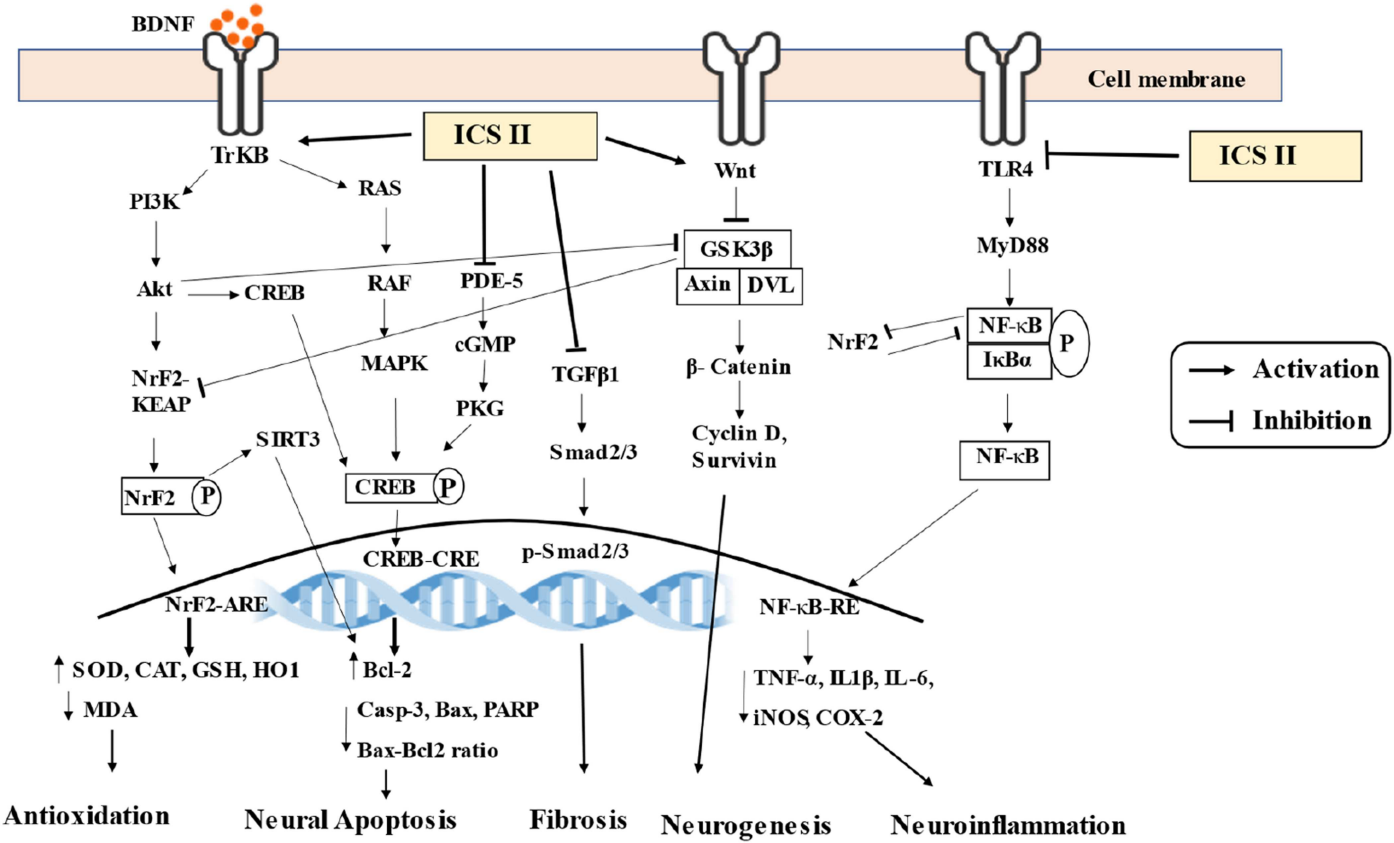
Schematic representation of different signalling pathways and their crosstalk regulated by icariside II (ICS II). Key signalling pathways modulated by ICS II include BDNF/TrkB/PI3K/Akt/CREB, RAS/RAF/MAPK, PDE‐5/cGMP/PKG, Wnt/β‐catenin, TGF‐β1/Smad, Keap1/Nrf2, and TLR4/MyD88/NF‐κB. By coordinated regulation of transcriptional elements (CREB‐CRE, Nrf2‐ARE, Smad‐binding elements, and NF‐κB‐responsive elements), ICS II promotes antioxidant effects, neurogenesis, suppresses neuroinflammation, attenuates fibrosis, and inhibits neuronal apoptosis.

The research findings indicate that ICS II exerts neuroprotective effects through the coordinated regulation of multiple interconnected signaling pathways mentioned in Figure 4. These pathways collectively modulate oxidative stress, neuroinflammation, mitochondrial dysfunction, apoptosis, fibrosis, and neurogenesis, thereby disrupting the interconnected pathological processes underlying various neurological disorders. Oxidative stress is considered a key pathological event that initiates a cascade of mitochondrial dysfunction, inflammatory activation, and neuronal apoptosis. ICS II activates the Nrf2/ARE signaling axis directly and through the Nrf2/SIRT3 pathway, promoting the transcription of antioxidant enzymes, including HO‐1, SOD, CAT, and GSH, while reducing lipid peroxidation. Restoration of redox homeostasis limits excessive ROS generation, which restores mitochondrial function and decreases oxidative damage. Additionally, through reducing oxidative stress, ICS II suppresses activation of the TLR4/MyD88/NF‐κB signaling pathway, leading to decreased production of pro‐inflammatory mediators such as TNF‐α, IL‐1β, IL‐6, iNOS, and COX‐2. The reduction in oxidative stress and inflammation subsequently influences apoptotic signaling. Specifically, activation of the BDNF/TrkB/PI3K/Akt/CREB and cGMP/PKG/CREB pathways promotes CREB‐mediated expression of anti‐apoptotic proteins, including Bcl‐2, while downregulating pro‐apoptotic factors such as Bax, caspase‐3, and PARP. Since mitochondrial oxidative injury is a main triggering factor of intrinsic apoptosis, the antioxidant actions of ICS II further promote neuronal survival by preventing mitochondrial dysfunction and limiting apoptotic cascade activation. In parallel, ICS II promotes endogenous repair mechanisms by activating Wnt/β‐catenin signaling through inhibition of GSK‐3β, thereby increasing the expression of Cyclin D1 and Survivin to facilitate neuronal proliferation and neurogenesis. In addition, inhibition of TGF‐β1/Smad signaling suppresses fibrotic remodeling, which may contribute to improved tissue repair. Collectively, these pathways promote the anti‐oxidation, anti‐inflammatory and anti‐apoptotic effects of ICS II, suggesting neuronal recovery through the combined preservation of neuronal integrity and activation of endogenous repair mechanisms. The findings indicate that ICS II functions as a multi‐target neuroprotective agent.

### 5.6 Conclusion

The preclinical evidence studies show that ICS II exhibits therapeutic effects in different experimental models of neurological disorders, mainly AD, PD, Cerebral ischemia and other neurological conditions such as neuroinflammation, neuropathic pain, diabetic neuropathy, and neurotoxicity. chronic cerebral hypoperfusion injury and Schizophrenia via regulating multiple signaling mechanisms of apoptosis, oxidative stress and neuroinflammation. ICS II has been reported to possess multitargeted therapeutic potential through the modulation of key molecular pathways and their associated mechanisms. Notably, the activation of PI3K/Akt and BDNF/TrkB cascades facilitates neuronal survival, synaptic plasticity, neurogenesis, and decreases apoptosis. Simultaneously, activation of the Keap1/Nrf2 pathway promotes the endogenous antioxidant defense system, effectively reducing oxidative stress. Furthermore, Suppression of NF‐κB signaling by ICS II reduces the release of pro‐inflammatory cytokines, thereby decreasing neuroinflammation. Additionally, regulation of the cGMP/PKG/CREB pathway decreases apoptosis, enhances mitochondrial function and contributes to cognitive improvement. Collectively, these experimental findings highlight the therapeutic promise of ICS II as a multitarget neuroprotective candidate. However, the current evidence is based exclusively on preclinical studies, and its therapeutic potential requires validation through well‐designed clinical investigations before its clinical applicability can be established.

### 5.7 Translational and Future Perspective

Preclinical evidence from both in vivo and in vitro studies indicates that ICS II demonstrates promising neuroprotective activity against different neurological disorders. However, despite these findings, no clinical studies have yet evaluated its therapeutic efficacy and safety in patients with neurological disorders, thereby limiting the translation of these experimental outcomes to human populations. In addition, the therapeutic efficacy of ICS II in neurological disorders is affected by pharmacokinetic limitations such as poor aqueous solubility, extensive first‐pass metabolism, and restricted systemic availability, resulting in decreased bioavailability. Hence, the future investigations should focus on pharmacokinetic optimization using advanced drug delivery systems, such as polymeric or lipid‐based nanoparticles, liposomes, nano emulsions, cyclodextrin inclusion complexes, or other formulation approaches that improve solubility, stability and brain delivery. In addition to this, pharmacokinetic and biodistribution studies are also required to characterize BBB penetration, CNS exposure, metabolic fate, and optimal dosing regimens under both physiological and pathological conditions. Furthermore, well‐designed clinical translation studies are required to establish the efficacy, long‐term safety, and therapeutic potential of ICS II in neurological disorders. Such investigations will be crucial for translating the promising preclinical findings of ICS II into a clinically viable neuroprotective therapy.

## Author Contributions

**Ashutosh Solanki:** conceptualization. **Khushi Sharma:** writing – original draft. **Jagrati Jain:** writing – original draft. **Niraj Kumar Singh:** writing – review and editing; supervision. **Praveen T Krishnamurthy:** supervision, writing – review and editing.

## Funding

The authors have nothing to report.

## Conflicts of Interest

The authors declare that they have no known competing financial interests or personal relationships that could have appeared to influence the work reported in this paper.

## Data Availability

Data sharing not applicable to this article as no datasets were generated or analysed during the current study.
